# Composite Discordant States and Quantum Darwinism

**DOI:** 10.3390/e24111509

**Published:** 2022-10-22

**Authors:** Eoghan Ryan, Mauro Paternostro

**Affiliations:** Centre for Quantum Materials and Technologies, School of Mathematics and Physics, Queen’s University, Belfast BT7 1NN, UK

**Keywords:** quantum to classical transition, objectivity, Quantum Darwinism, quantum correlations

## Abstract

The framework of Quantum Darwinism strives at characterizing the quantum-to-classical transition by introducing the concept of redundancy of information—as measured by Mutual Information—that a set of observers would acquire on the state of a physical system of interest. Further development on this concept, in the form of Strong Quantum Darwinism and Spectrum Broadcast Structures, has recently led to a more fine-grained identification of the nature of such information, which should not involve any quantum correlations between observing and observed systems, while the assessment of information proliferation from individual systems has attracted most of the attention so far, the way such mechanism takes place in more complex states is open to exploration. To this end, we shall consider a two-qubit state, sharing initial quantum correlations in the form of Quantum Discord, and different dephasing-like interactions between them and an observing environment. We will focus on the amount of information regarding the subsystem not involved in the interaction that is proliferated to the environment. We shall refer to this as mediated redundancy. We will show that, in some cases, the channel capacity of the subsystems, given these interactions, can exceed that of the fragments.

## 1. Introduction

Among the frameworks striving at the provision of a fundamental explanation of the mechanism driving the quantum-to-classical transition, Quantum Darwinism takes a significant standing in that it promotes the environment affecting the dynamics of a system of interest as an active part of the process [1,2,3,4,5,6,7,8,9,10,11,12,13,14,15,16,17,18,19,20,21]: by monitoring the state of the system, a multi-observer environment would acquire information on the latter. The proliferation of redundant—that is, consistent among the various observers—information signals the emergence of classicality, made *objective* by the mutual agreement among the observing parties. The concept of *Strong Quantum Darwinism* has then refined such an idea by severely constraining the nature of the information that the observers should have at hand, which should only be classical. Furthermore, the idea of strong inter-independence, i.e., a lack of correlations between the environmental fragments, led to the introduction of *Spectrum Broadcast Structures* [22,23,24], which limits the types of states that will allow the emergence of completely classical information.

Quantum discord [25,26] was introduced to characterize, both qualitatively and quantitatively, the quantum information shared by the parties of a composite state when considering the maximum achievable amount of classically observable information. A potential link between Quantum Discord and non-Markovianity was explored in Ref. [27]. It was suggested that, given an initially discordant state, the reduced dynamics could not be completely positive, thus leading to the formulation of a measure of non-Markovianity [28]. It has been later found [29,30] that Quantum Discord plays no role in the characterization of a map from such viewpoint, thus suggesting its irrelevance in the dynamical emergence of non-Markovianity. However, it is well-known that the set of zero-discord states is nowhere dense [31] while, for a finite time interval, a Markovian process is incapable of taking (and keeping) a state outside of the set of zero-discord states into said set. Specifically, the number of times it can intersect with said set is upper bounded by the number of eigenvalues in the map. This means that initially discordant states could, in fact, be taken to zero-discord states, as the initial value of Quantum Discord is not linked to the emergence of non-Markovianity.

As the initial system-environment state will have no discord, this does not immediately appear to be of interest. What will be relevant in our study is how the presence of initial Quantum Discord in the state of the system will affect the way information on the latter is to be proliferated into the environment.

In this paper, we are interested in exploring how the presence of initial Quantum Discord in a composite system affects the way information proliferates into an environment. To our knowledge, the only other work done on a composite system is Ref. [32]. However, this covered only the case of a system in which its two subsystems were prepared in a pure, uncorrelated state. As such we see our work as a natural complement in the question of composite-state redundancy. Moreover, it will be interesting to see whether, in light of the the emergence of redundancy, the system would retain its interior quantum correlations.

Given a classical-quantum state [33] of the form

(1) $$\rho_{AB}=\sum_{k}p_{k}|i_{A}^{k}\rangle \langle i_{A}^{k}|⊗\rho_{B}^{k}$$

the states $\rho_{B}^{k}$, even if pure, can have non-orthogonal support, ie $\rho_{B}^{i}\rho_{B}^{k}\neq 0,i\neq k$. For such a state as (1), the only way to obtain any measure of the Quantum Discord is to perform a measurement on subsystem B. Given this, even if one of the subsystems of this system possesses states that can be encoded easily into the environment, the other subsystem could have states which have overlap with the set of states that commute with the reduced dynamics for that subspace. Moreover, the majority of states fall into this category, which means that perfect Strong Quantum Darwinism seems unlikely for all but the most limited class of states. Due to this, interactions with only one part of the subsystem may be crucial to whether more or less information can be encoded about the whole.

The remainder of this paper is organized as follows. In Section 2, we briefly review the concepts of Quantum Darwinism, Strong Quantum Darwinism, and Quantum Discord. Section 3 introduces our choice of system, environment and the interaction models that we wish to investigate, illustrating the behavior of the various figures of merit discussed previously. In Section 4, we illustrate our findings of how these different interactions on different subsystems can mediate different levels of Quantum Darwinism, Strong Quantum Darwinism and Quantum Discord. Section 5 will summarise our results and highlight the questions that remain to be addressed.

## 2. Overview of Relevant Figures of Merit

Quantum Darwinism promotes the environment that interacts with the system of interest to the active role of a (in general composite) dynamical object that acquires information on the system over time [1,2,3,4,5,6]. The basis of election of such process are called *pointer states* [34]. They are the eigenvectors of the operator upon which the environment acquires information that are left unaffected by the system-environment interaction, and are selected via the interaction with the environment by the process of *einselection* [34]. Consider an initially uncorrelated system-environment state such as

(2) $${|\psi \left(0\right)\rangle }_{SE}=\left(\sum_{i}k_{i}{|s_{i}\rangle }_{S}\right)⊗{|e_{0}\rangle }_{E}.$$

Here, $\sum_{i}k_{i}{|s_{i}\rangle }_{S}$ is the initial state of the system, expanded over the pointer-state basis $\left\{{s_{i}\rangle }_{S}\right\}$, and $|e_{0}\rangle_{E}$ is the initial state of a single-element environment. After the joint system-environment interaction, the environment becomes correlated with the pointer states as

(3) $${|\psi \left(t\right)\rangle }_{SE}=\sum_{i}k_{i}|s_{i}\rangle_{S}⊗{|e_{i}\rangle }_{E}.$$

Consider now a multiple-element environment, whose components $E_{j}$ are mutually independent entities. If the process above results in an an information-proliferation mechanism giving rise to the branching structure

(4) $${|\psi \left(t\right)\rangle }_{SE}=\sum_{i}k_{i}|s_{i}\rangle_{S}\underset{j}{⨂}{|e_{i}\rangle }_{E_{j}}.$$

the information brought about by the various environmental elements is *redundant* in that different environmental fragments mutually agree on the amount of information they have on the system, the framework of Quantum Darwinism would claim the completion of the process of classicalisation of the state of the system. The figure of merit through which this is witnessed is the Mutual Information

(5) $$I\left(S:F_{k}\right)=H{\left(S\right)}_{\rho }+H{\left(F_{k}\right)}_{\rho }-H{\left(SF_{k}\right)}_{\rho },$$

which quantifies the total correlations between the system *S*, prepared in state $\rho_{S}={\mathrm{tr}}_{F_{k}}\left(\rho_{SF_{k}}\right)$ and the environmental fraction $F_{k}$, whose state we call $\rho_{F_{k}}={\mathrm{tr}}_{S}\left[\rho_{SF_{k}}\right]$. We have introduced the joint *S*-$F_{k}$ state $\rho_{SF_{k}}$ and the von Neumann entropy $H\left(\sigma \right)=-\mathrm{tr}\left[\sigma \log_{2}\sigma \right]$ of a generic density matrix σ. We have also used the notation $H{\left(A\right)}_{\sigma }\equiv H\left(\sigma_{A}\right)$, and analogues, when referring to the entropy of the quantum state $\sigma_{A}$ of a given system *A*. As, in the remained of the paper, we will always refer to quantum entropies, we will henceforth drop the pedex ρ and thus consider $H\left(A\right)\equiv H{\left(A\right)}_{\rho }=H\left(\rho_{A}\right)$, unless otherwise specified.

**Figure 1:**
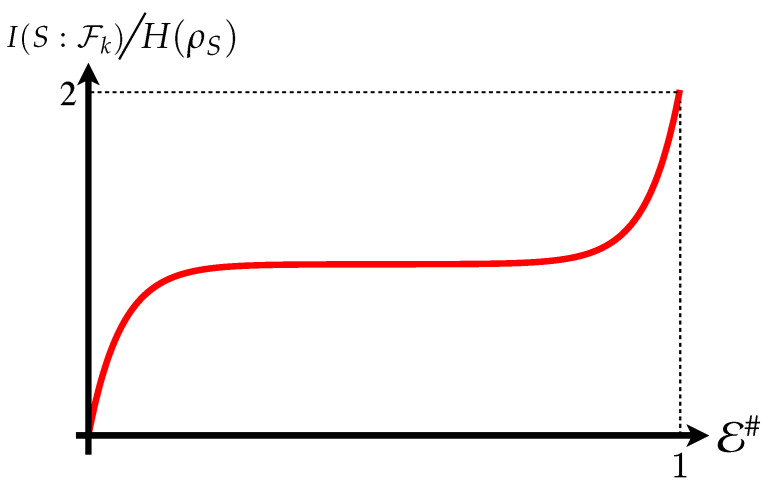
The Mutual Information plateau that emerges when full redundancy of the systems information is achieved. Further fragments reveal no information, with the sharp rise at the end attributed to the quantum correlations only available with the whole environment. The curved rise, as opposed to the sharp edges observed in all the other plots in this work, occurs when the smallest constituents of the environment are incapable of encoding all information regarding the system.

If redundancy occurrs, Mutual Information would not depend on the size $E^{\#}$ of the environmental fraction $F_{k}$: increasing it will not result in additional information on *S* encoded in the environment. This gives rise to a *partial information plateau*, where $I(S:F_{k})$ would in fact remain unchanged against the dimension of $F_{k}$. Once this occurs, the only way to further increase mutual information is for $F_{k}$ to coincide with the whole environment, which will lead to $I\left(S:F_{k}\right)=2H\left(S\right)$, as seen in Figure 1.

In Ref. [25], a disparity between two possible definitions of Mutual Information was pointed out, which, while classically identical, become dissimilar in the quantum regime. Due to Bayes rule, the following chain of identities for the Mutual Information of two classical random variables *X* and *Y* holds

(6) $$\begin{array}{cc} I(X:Y) & =H(X)+H(Y)-H(X,Y)=H(X)-H(X|Y)=H(Y)-H(Y|X). \end{array}$$

Here H(X,Y) is the joint entropy and H(X|Y) [or H(Y|X)] the conditional entropy, which are evaluated from the knowledge of the single- and joint-variable probability distributions p(Y=y) [p(X=x)] and p(X=x,Y=y). However, for quantum Mutual Information, the quantities

(7) $$I\left(S:F_{k}\right)=H\left(S\right)+H\left(F_{k}\right)-H\left(SF_{k}\right)$$

and

(8) $$I\left(S:F_{k}\right)=H\left(F_{k}\right)-H\left(S|Fk\right)$$

are not equivalent. Here, we have used the notation $H{\left(A\right)}_{\rho }\equiv H\left(\rho_{A}\right)$, and analogues, when referring to the entropy of the quantum state $\rho_{A}$ of a given system *A*. As, in the remained of the paper, we will always refer to quantum entropies, we will henceforth drop the pedex ρ and thus consider $H\left(A\right)\equiv H{\left(A\right)}_{\rho }=H\left(\rho_{A}\right)$, unless otherwise specified. The inequivalence between Equations (4) and (5) arises from the definition of quantum conditional entropy $H(S|F_{k})$ as the information that can be acquired on $\rho_{S}$ *given* some state $\rho_{F_{k}}$. This requires the use of a measurement process. For quantum states, knowledge of one system will increase or decrease the information one has of the other. Along with Refs. [22,35], the classical nature of the information proliferated to $F_{k}$, as well as its objective nature via redundancy, may also need to be taken into account, giving rise to Strong Quantum Darwinism. We thus arrive at the the Asymmetric Mutual Information

(9) $$J\left(\hat{S}:F_{k}\right)=H\left(\hat{S}\right)+H\left(F_{k}\right)-H\left(\hat{S}F_{k}\right),$$

where the symbol $H(\hat{S})$ stands for the entropy of the state ${\hat{\rho }}_{S}$ of a given state $\rho_{S}$ of system S that has been acted upon by a positive operator values measurement (POVM) of elements $\left\{\Pi_{i}\right\}$. Equation (9) quantifies the information available given a measurement on one part of the composite system has occurred. In this respect, determining whether or not the state of the non-queried system is classical is vital to determine the emergence of classicality as per the Darwinistic paradigm. It is important to note that Equation (9) is asymmetric in nature, i.e., $J\left(\hat{S}:F_{k}\right)\neq J\left(S:{\hat{F}}_{k}\right)$. As the measurements being invoked can be viewed as a local map on the queried subsystem, by the monotonicity of the relative entropy, the Asymmetrical Mutual Information is upper bounded by the Mutual Information. This then leads to the definition of Quantum Discord

(10) $$D\left(\hat{S}:F_{k}\right)=I\left(S:F_{k}\right)-J\left(\hat{S}:F_{k}\right).$$

This quantity encompasses quantum correlations beyond classical ones that exist between quantum states [25,36]. As noted in Ref. [26], it shares a complementary role with Equation (9) and is upper bounded by the Mutual Information. A comprehensive review of quantum discord and its role in quantum information science is given in Ref. [37]. Due to the concave nature of the conditional entropy, and the convex nature of a POVM, rank-one projectors maximise the Asymmetrical Mutual Information, giving us the Holevo Information [26] (which we write using the density matrix-dependent notation to avoid any ambiguity)

(11) $$\chi \left(\hat{S}:F_{k}\right)=\max_{\Pi_{i}}\left[H\left(\rho_{F_{k}}\right)-\sum_{i}p_{i}H\left(\rho_{F_{k}}^{i}\right)\right],$$

where $\rho_{F_{k}}^{i}$ is the state of the fragment conditioned on the state of the system, and $p_{i}$ is the probability associated with the measurement result of the projector $\Pi_{i}$. This value upper bounds the *classical* capacity of a given channel, and is henceforth referred to as the channel capacity of a given state.

## 3. Description of the Model

We will consider a system comprising two qubits in the following separable, yet discordant state

(12) $$\rho_{s}={p|0\rangle \langle 0|}_{1}⊗{\left|0\rangle \langle 0\right|}_{2}+{\left(1-p\right)|1\rangle \langle 1|}_{1}{⊗|+\rangle \langle +|}_{2},$$

where $|+\rangle =(|0\rangle +|1\rangle )/\sqrt{2}$ is the eigenstate of the *x* Pauli operator $\sigma_{S}^{x}$ with eigenvalues +1. Here, {|0〉,|1〉} are the eigenstates of the *z* Pauli matrix $\sigma^{z}$, which we take as the elements of the computational basis for each particle involved in the problem. As shown in Ref. [33], the state in Equation (12) can be created from a classical state located in the set of zero-discord states using only local maps. In our calculations, we will set p=1/2, which means global measurements permit no bias.

As mentioned above, we will consider the case of a bipartite system comprising qubits $s_{1}$ and $s_{2}$ prepared, in general, in a quantum correlated state. On the other hand, the $i^{\mathrm{th}}$ environmental fragment is prepared in the eigenstate of the *x* Pauli operator $\sigma_{i}^{x}$ with eigenvalues +1, that is ${|e\rangle }_{i}=\left({|0\rangle }_{i}+{|1\rangle }_{i}\right)/\sqrt{2}$. As we will see shortly, this ensures that the preparation of the environmental fragments does not involve states that commute with the eigenvectors of the Hamiltonian of interaction between said fragment and the system [3]. In fact, such mechanism is taken to be of a form that results in pure dephasing on the state of the system (or individual elements of the environment). The question on which form of system-environment interaction is capable of letting the Darwinistic phenomenology emerge most effectively has been the focus of some recent investigations. In Ref. [38] it has been shown that one can inhibit Darwinistic behaviour of Mutual Information by simply tuning the system-environment interaction from a dephasing-like to an energy exchange model. This motivates our choice of interaction models as dephasing-like ones, which are the most favourable for the phenomenology that we aim at investigating. We have taken an environment consisting of three elements for easiness of computation. This is the smallest dimension that will enable the emergence of a partial information plateau (PIP), establishing an upper bound to the size of the environmental fraction at which the "knee" of such plateau occurs: a larger environment will change the position of the knee, which would move to smaller environmental fractions, at the same time lessening the sharpness of the changes in behaviour of the figures of merit addressed in our study. The number of elements was kept the same for all the calculations performed in the manuscript.

**Figure 2:**
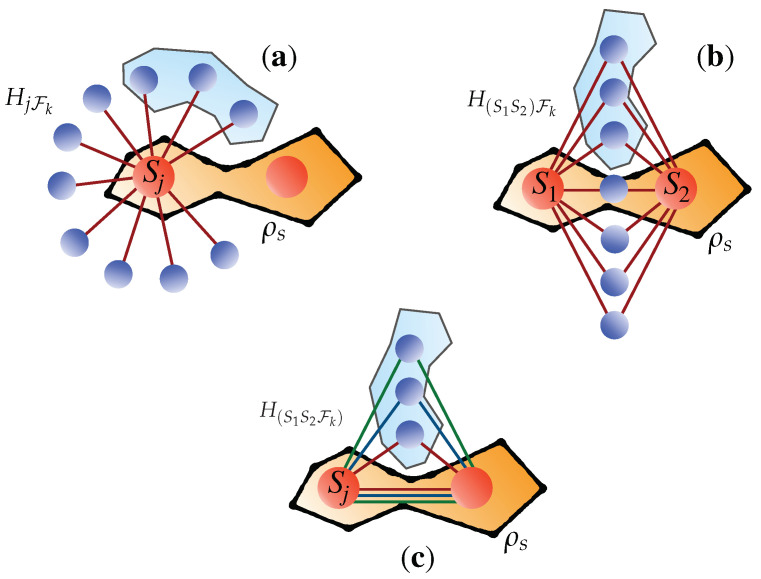
Illustration of the physical configurations implied by the Hamiltonian models in Equation (13) [panel (**a**)], Equation (14) [panel (**b**)] and Equation (15) [panel (**c**)]. Solid straight lines represent Hamiltonian coupling. The subsystems $S_{1}$ and $S_{2}$ are prepared in the discordant state $\rho_{S}$ while each of the environmental elements in |e〉.

The various configurations that will be considered are [cf. Figure 2]

1. The coupling between qubit $S_{j}$ (j=1,2) of the bipartite system and the environment. The interaction model that we consider in this case is
  (13) $$H_{jF_{k}}=J\sigma_{S_{j}}^{z}⊗\sum_{i}\sigma_{i}^{z}\;\;\;\left(j=1,2\right).$$
2. The simultaneous, yet individual coupling of the system qubits with the elements of the environment. The model to consider in this case reads
  (14) $$H_{\left(S_{1}S_{2}\right)F_{k}}=J\left(\sigma_{S_{1}}^{z}+\sigma_{S_{2}}^{z}\right)⊗\sum_{i}\sigma_{i}^{z}$$
3. The simultaneous (three-body) interaction of both the system’s qubits and the $i^{\mathrm{th}}$ element of the environment. This coupling would be described by the Hamiltonian
  (15) $$H_{\left(S_{1}S_{2}F_{k}\right)}=J\sum_{i}\sigma_{S_{1}}^{z}⊗\sigma_{S_{2}}^{z}⊗\sigma_{i}^{z}$$

Here *J* is the strength of the respective coupling and $\sigma^{z}$ is the *z* Pauli matrix. As all coupling Hamiltonians commute with such operator, we neglect the free part of the Hamiltonian of the system. As Equations (13)–(15) consist of mutually commuting terms, each interaction results in a local unitary between the system and coupled environmental element.

These models will allow us to explore how well the information of the other, non-interacting system will be proliferated, given an interaction restricted to one subsystem. This proliferation should depend on the strength and type of correlations that already exist between the two subsystems. In this case, the states of the subsystem are mixed states, which can be encoded in the environment [3]. The discordant nature of the state could affect the way information regarding $\rho_{S_{1}}$ will be proliferated into the environment. We will thus be interested in characterizing the way the existing discord will affect this mediated encoding of the non-interacting subsystem into the environments and how this will affect the contribution of Holevo Information and Quantum Discord to the Mutual Information.

In the following assessment of the seeding of redundant information in the environment, we will study the effects of a growing coupling strength over the temporal window within which we would study evolution. In the following Sections, any time the label for a subsystem is used in one of the figures of merit at the center of this study, it should be understood that the corresponding state has been evolved according to the model under consideration. Such evolution is implemented by solving the Schrödinger equation propagating in time the initial system-environment state and considering the reduced system-fraction state achieved by tracing out part of the environment.

## 4. Results

### 4.1. $S_{1}$ Coupled to the Environment

In this Section, we address the possibility to achieve redundant encoding, and study the nature of the information stored in the state of the environment, when considering the model in Equation (13) for j=1. In the remainder of our investigation, the initial state of the system is taken to be as stated in Equation (12). In what follows, the process of proliferation of information would be considered as redundant when occurring up to a small deficit of <1%.

**Figure 3:**
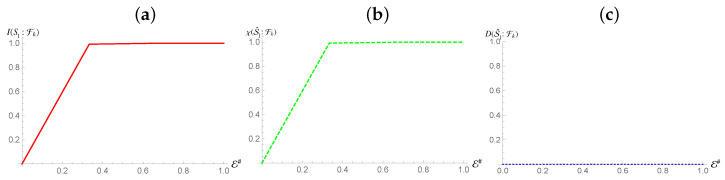
Panel (**a**,**b**): Mutual information (Holevo information) between subsystem $S_{1}$ and and environmental fraction $F_{k}$ for the Hamiltonian model in Equation (13). Panel (**c**): Quantum Discord between the environmental fragments $F_{k}$ and $S_{1}$ for the same Hamiltonian model as in panels (**a**,**b**). In all panels, the horizontal axis shows the size of the environment $E^{\#}$, while for all the simulations considered we have taken Jt=10 with *t* the evolution time. The horizontal axis on all graphs is in terms of the Mutual Information normalized using the systems of interests entropy.

Ref. [3] states that a maximally mixed state can still be encoded redundantly into an environment, provided the environmental elements can increase their entropy appropriately to store the information of the monitored system. The only deviation from the usual redundancy plots will be the absence of the sharp rise resulting from taking the full environment [cf. Figure 1]. Figure 3 shows the behavior of Mutual Information and Holevo Information against the size of the environmental fraction $E^{\#}$ being considered for the preparation of the system stated in Equation (12), while both show a typical *redundant encoding* behaviour, their quantitative equivalence demonstrates that all information stored is classical in nature, and represents the total information about $S_{1}$. This means that information about the complementary states to the pointer states is absent even for global measurements of the system. Furthermore, this tells us that the channels acting between this subsystem and the fragments are not capable of generating Quantum Discord [33,39].

**Figure 4:**
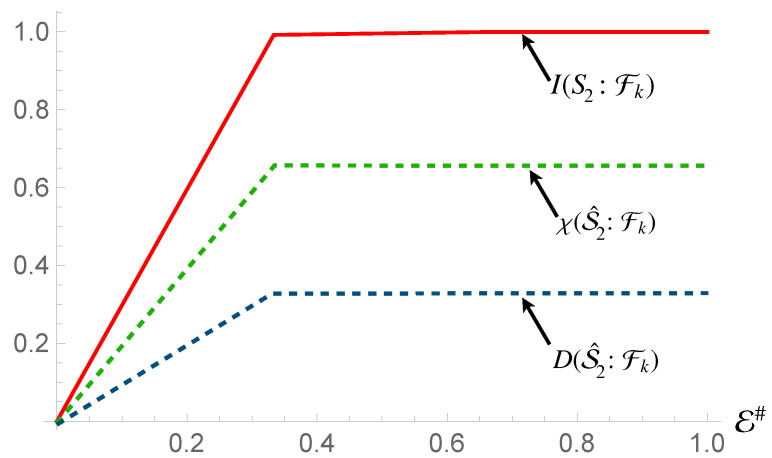
Mutual information, Holevo information and Quantum Discord between subsystem $S_{2}$ and and environmental fraction $F_{k}$ for the Hamiltonian model in Equation (13). The horizontal axis shows the size of the environment $E^{\#}$, while for all the simulations considered we have taken Jt=10 with *t* the evolution time. The horizontal axis is in terms of the Mutual Information normalized using the systems of interests entropy.

In contrast, we see the opposite behaviour for $S_{2}$ [cf. Figure 4], while we once again see the emergence of a redundancy in the behavior of Mutual Information, thus showing that a mediation by quantum correlation is sufficient to propagate redundant encoding of information to a system that is not directly coupled to the environment, such information is not entirely classical: quantitatively, the Holevo Information is only a fraction δ of the von Neumann entropy of the state of the system, with

(16) $$\delta =\frac{I\left(S_{2}:F_{k}\right)-\chi \left({\hat{S}}_{2}:F_{k}\right)}{H(S_{2})}.$$

This means that Strong Quantum Darwinism and Spectrum Broadcast Structures are not compatible with this arrangement, (more accurately, Strong Quantum Darwinism can only be said to hold for a fraction δ of the entropy of the state of the system). Due to the non-orthogonal nature of the conditional states of $S_{2}$, Quantum Discord is present with respect to such subsystem. The observed value of Quantum Discord is identical to the value for the initial state of the system when performing measurements on $S_{2}$. This is exactly what we would expect, as the state itself is invariant with respect to the interaction between the first subsystem and the fragments. Due to this, given $S_{1}$ is correlated with the conditional states of the fragments, they now share the same Quantum Discord with $S_{2}$.

These results raise the question of how the initial correlations between $S_{1}$ and $S_{2}$ affect or influence the correlations between the second subsystem and the environment, when the first subsystem is mediating the interaction with the environment. First, as shown in Appendix A [cf. Equation (A1)], the Mutual Information between $S_{2}$ and the environmental fraction $F_{k}$ is upper bounded by the original Mutual Information present between subsystems $S_{1}$ and $S_{2}$, i.e.

(17) $$I\left(S_{2}:S_{1}\right)\geq I\left(S_{2}:F_{k}\right).$$

This holds for asymmetrical Mutual Information as well, as this measure is achieved via a local set of measurements on $S_{2}$, and as such will also upper bound the measure obtained via the action of discarding a subsystem, due to the monotonicity of the relative entropy

(18) $$\chi \left({\hat{S}}_{2}:S_{1}\right)\geq \chi \left({\hat{S}}_{2}:F_{k}\right).$$

Furthermore, due to Equation (17), we have

(19) $$I\left(S_{2}:S_{1}\right)\geq \chi \left({\hat{S}}_{2}:F_{k}\right)\;\mathrm{and}\;I\left(S_{2}:S_{1}\right)\geq D\left({\hat{S}}_{2}:F_{k}\right).$$

Therefore, the initial Mutual Information between the subsystems provides an upper bound to both the Holevo Information and Quantum Discord between $S_{2}$ and environmental fragments of any size, regardless of whether the measurement needed to evaluate the Holevo Information takes places on the subsystem or the fragment. This is an interesting point, as it implies that pre-existing correlations upper bound the redundancy of the information of one subsystem, given an interaction with the other.

As the difference $I\left(S_{2}:S_{1}\right)-\chi \left({\hat{S}}_{2}:S_{1}\right)$ is invariant under unitary operations on the joint state of $S_{1}$ and $F_{k}$— they would entail local operations for the $S_{2}$–$S_{1}$ bipartition—and both these figures of merit are monotonic under partial trace—one would be tempted to conclude that

(20) $$D\left({\hat{S}}_{2}:S_{1}\right)\geq D\left({\hat{S}}_{2}:F_{k}\right).$$

This, however, is not the case, as there is no guarantee that, for arbitrary systems, the Mutual Information and Holevo Information will decrease by the same amount under the action of the partial trace. However, an explicit calculation shows that the Quantum Discord between $\rho_{S_{2}}$ and the environmental fragments being considered is upper bounded by the initial value for the Quantum Discord between the two subsystems for all the mediated, local interactions. It is worth noting that, due to Equations (17) and (18), while $D\left({\hat{S}}_{2}:S_{1}\right)\geq D\left({\hat{S}}_{2}:F_{k}\right)$ does not necessarily hold, the higher $D({\hat{S}}_{2}:S_{1})$, the tighter the upper bound on $\chi ({\hat{S}}_{2}:F_{k})$.

**Figure 5:**
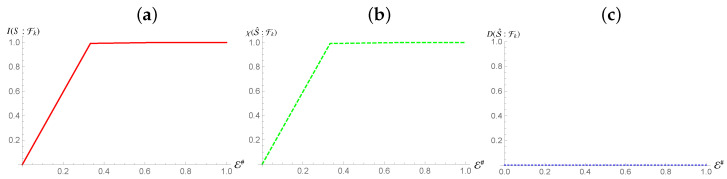
Panel (**a**,**b**): Mutual information (Holevo information) between the whole system and and environmental fraction $F_{k}$ for the Hamiltonian model in Equation (13). Panel (**c**): Quantum Discord between the environmental fragments $F_{k}$ and the whole system for the same Hamiltonian model as in panels (**a**,**b**). In all panels, the horizontal axis shows the size of the environment $E^{\#}$, while for all the simulations considered we have taken Jt=10 with *t* the evolution time. The horizontal axis on all graphs is in terms of the Mutual Information normalized using the systems of interests entropy.

Lastly, we look at the phenomenology of redundancy when the whole compound system is considered. It displays the same behaviour as $S_{1}$, with Figure 5 showing that measurements consisting of composite, local measurements on $S_{1}$ and $S_{2}$, can perfectly distinguish the states that result from the overall interaction. Therefore, the Mutual Information is equal to the Holevo Information. In this case, given an interaction between the subsystem that allows full access to all information via Holevo Information and the environment, it is possible to satisfy Strong Quantum Darwinism. As the following inequality holds

(21) $$\chi \left({\hat{S}}_{1},{\hat{S}}_{2}:F_{k}\right)\geq \chi \left({\hat{S}}_{1}:F_{k}\right),$$

due to the monotonicity of the relative entropy under the action of the partial trace map, we see that the Holevo Information shared by the compound system with an environmental fragment is lower-bounded by the analogous quantity shared by any of of the subsystems and said fragment.

Recently, it has been shown [40,41] that the accessible information of the system, or its channel capacity, is just as important a metric to consider as that of fragments. The quantification of such a figure of merit involves measurements on the environment, as opposed to the system, which is more in line with what one would expect when querying redundant information in an environment. Maximisation now occurs over the set of rank-1 projectors performed on the elements of the considered fragment, which gives the quantity $\chi (S:{\hat{F}}_{k})$, with straightforward interpretation of the notation being used. Moreover, as shown for Spectrum Broadcast Structures, $\chi (\hat{S}:F_{k})$ and $\chi (S:{\hat{F}}_{k})$ would have to be equivalent to both the Mutual Information and each other in order for such a structure to emerge. It is worth noting that, when $\chi \left(\hat{S_{i}}:F_{k}\right)=I\left(S_{i}:F_{k}\right)$, the state ρ that is under consideration must take the form [22]

(22) $$\rho =\sum_{k}p_{k}|s_{S}^{k}\rangle \langle s_{S}^{k}|⊗\rho_{F}^{k}.$$

This is described as the state resulting from “good decoherence” [3], and as such can already be seen as a state measured in the pointer basis. Due to this, as a result of the data processing inequality, we find

(23) $$\chi \left(\hat{S_{i}}:F_{k}\right)\geq \chi \left(S_{i}:{\hat{F}}_{k}\right).$$

The reverse can easily be seen to also hold for cases when $\chi \left(S_{i}:{\hat{F}}_{k}\right)=I\left(S_{i}:F_{k}\right)$. Therefore, for a state satisfying Strong Quantum Darwinismin the manner described above, the channel capacity of the system will always be upper bounded by that of the fragments.

**Figure 6:**
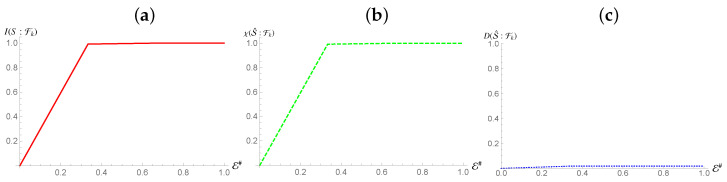
Panel (**a**,**b**): Mutual information (Holevo information) between the whole system and environmental fraction $F_{k}$ for the Hamiltonian model in Equation (13), this time given measurements on the environment. Panel (**c**): Quantum Discord between the environmental fragments $F_{k}$ and the whole system for the same Hamiltonian model as in panels (**a**,**b**), this time given measurements on the environment. In all panels, the horizontal axis shows the size of the environment $E^{\#}$, while for all the simulations considered we have taken Jt=10 with *t* the evolution time. The horizontal axis on all graphs is in terms of the Mutual Information normalized using the systems of interests entropy.

The next step then is to determine whether the environment shares only classical information with the system. This would allow us to determine that the overall state satisfies the requirements for both Strong Quantum Darwinism and Spectrum Broadcast Structures [23,42]. Now, the projective measurements will be performed on the environmental fragments, as opposed to the system of interest. Here, we observe an interesting situation. In the case of $S_{1}$ and the whole system, the channel capacity of the system is identical to that of the environment, much as we observed for the first interaction. However, for $S_{2}$, we do not observe this. Looking at Figure 6, we see that in this case, the channel capacity of $S_{2}$ is higher than that of the fragments, with the Holevo Information from it matching almost completely the Mutual Information (with the exception of a small deficit).

### 4.2. $S_{2}$ Coupled to the Environment

**Figure 7:**
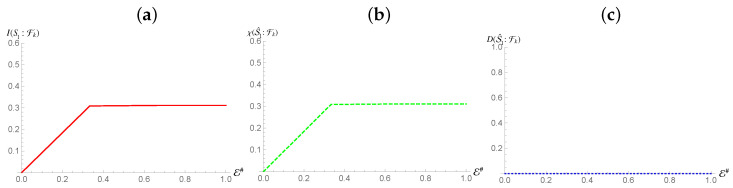
Panel (**a**,**b**): Mutual information (Holevo information) between $S_{1}$ and environmental fraction $F_{k}$ for the Hamiltonian model in Equation (13). Panel (**c**): Quantum Discord between the environmental fragments $F_{k}$ and $\rho_{S_{1}}$ for the same Hamiltonian model as in panels (**a**,**b**). In all panels, the horizontal axis shows the size of the environment $E^{\#}$, while for all the simulations considered we have taken Jt=10 with *t* the evolution time. The horizontal axis on all graphs is in terms of the Mutual Information normalized using the systems of interests entropy.

We now address the case of a coupling between $S_{2}$ and the environment, according to Equation (13) with j=2. This might seem a trivial difference with respect to the case studied in Section 4.1. However, there is an important asymmetry between the two cases due to the form of the initial state $\rho_{s}$ in Equation (12): at variance with the case studied previously, the reduced state of $S_{2}$ brings about *quantumness* in that ${\mathrm{tr}}_{1}\left(\rho_{S}\right)$ is non-diagonal in the computational basis. Therefore, the focus of the analysis here is the potential differences induced in the phenomenology of the encoding of information in the state of the environment by the non-classical nature of the state of the *mediator* $S_{2}$. Figure 7 shows that while an interaction on $S_{2}$ is still capable of mediating redundancy, it can only do it for a fraction of $H(\rho_{S_{1}})$. However, despite this deficit of redundancy, all of such information is classical, indicating that while perfect proliferation of the information is not possible, it is still classical in nature. This seems to indicate that the Quantum Discord present between the two subsystems prevents the full redundancy of the classical information of $S_{1}$. That being said, it still means that the classical information of $S_{1}$ is proliferated despite the non-zero Quantum Discord nature of the existing correlations it shares with the mediating system.

**Figure 8:**
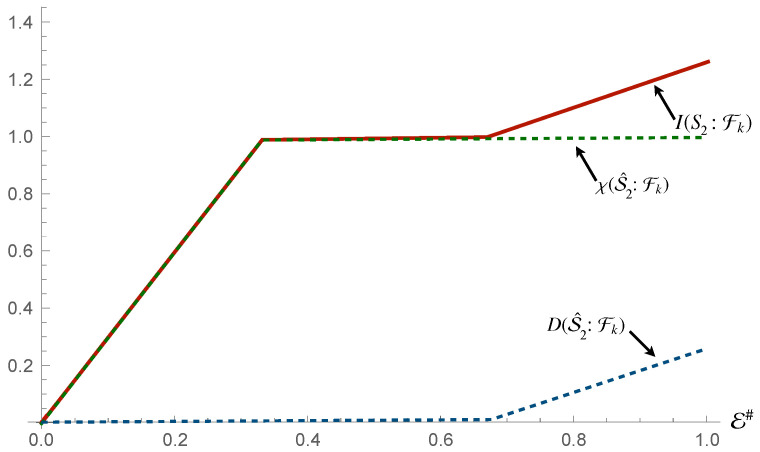
Mutual information, Holevo information and Quantum Discord between $S_{2}$ and environmental fraction $F_{k}$ for the Hamiltonian model in Equation (13). In all panels, the horizontal axis shows the size of the environment $E^{\#}$, while for all the simulations considered we have taken Jt=10 with *t* the evolution time.

As for $S_{2}$, we see a surprising behaviour: it still manages to proliferate all its information into the surrounding fragments, as it can be ascertained from Figure 8. Moreover, we can see that the degree of purity of the formed state is higher, due to the higher initial purity of the state of $S_{2}$. However, all such information is classical, while the rise of Mutual Information at the end can be attributed to additional information being encoded in the state of the environmental fragment made available only for a large enough size of the latter. This is likely due to the fact that, as stated in Ref. [3], a mixed state can still be redundantly encoded, and also explains the absence of Quantum Discord.

**Figure 9:**
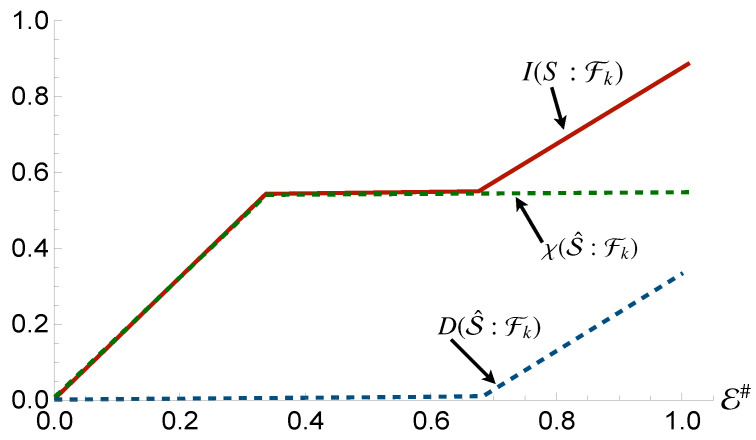
Mutual information, Holevo information and Quantum Discord between the whole system and environmental fraction $F_{k}$ for the Hamiltonian model in Equation (13). In all panels, the horizontal axis shows the size of the environment $E^{\#}$, while for all the simulations considered we have taken Jt=10 with *t* the evolution time.

Figure 9 shows the results associated with the assessment of the whole compound state: counter to the behaviour we observed for a single subsystem, we do not observe perfect redundancy of the mutual information, which reaches a plateau at ∼0.55H(S). This shows that, at the global level, given an interaction on the part of the state contributing to the presence of Quantum Discord, the full information of the system cannot be transferred into the fragments. However, all the information present is still completely classical in nature, indicating that this state still satisfies the requirements of Strong Quantum Darwinism, up to a certain deficit. Furthermore, alteration of the bias between the two non-orthogonal states in $S_{2}$ can affect the deficit by lowering or raising it.

It is also interesting that, despite an interaction with the non-classical part of the compound system, it is not possible to find quantum information redundantly encoded at any level apart from a global one, regardless of the fact that the measurement process now address the whole system. This would seem to indicate that the effective maps acting on the system and any part of the environment are not of the discord-creating nature.

Focusing on measurements on the environmental fragments instead of the subsystems, we observe that the values of Holevo Information and Quantum Discord shared with the fragments and either subsystems, or the whole system, are identical to those obtained if the system is measured. This indicates that, in the case of $S_{2}$, despite its contribution to Quantum Discord, is still capable of satisfying both Strong Quantum Darwinism and Spectrum Broadcast Structures, while the first subsystem can only be said to satisfy Strong Quantum Darwinism up to a certain deficit of the Mutual Information. The Quantum Discord between the subsystems, unlike in the case where $S_{1}$ is coupled to the environment, has again changed. Now, the Holevo Information for both of them is equal to the Mutual Information, meaning that the quantum information previously present has disappeared entirely, and that the map acting on $S_{2}$ is of the Discord Breaking variety. This indicates that the reduced dynamics that took place between the two subsystems is indeed non-Markovian [43].

### 4.3. Simultaneous Individual Couplings

We now address the case of simultaneous yet individual couplings of the subsystems with the environment, as described by the Hamiltonian model in Equation (14). The commuting nature of the two terms entering such models implies that the Mutual Information and Holevo Information of the subsystems is upper bounded by the values achieved for the individual interactions between the subsystems studied in Section 4.1 and Section 4.2. This can be seen as

(24) $$I\left(S_{i}:F_{k}\right)=D(\left(I_{i}⊗N_{k}\right)U_{i,k}\rho_{ik}U_{i,k}^{†}\left|\right|\left(I_{i}⊗N_{k}\right)U_{i,k}\left(\rho_{i}⊗\rho_{k}\right)U_{i,k}^{†})$$

where i=1,2 and *k* identifies the environmental fraction, N is the channel acting on $F_{k}$ due to the tracing out of the irrelevant subsystem, $I_{i}$ is the identity for subsystem *i* and $U_{i,k}$ is the unitary interaction controlling the joint evolution of said subsystem and fragment. Due to the unitary interaction being identical to that found in the previous two interaction models, and the monotonicity of the relative entropy under the action of a local channel, we have

(25) $$\begin{array}{c} D(\left(I_{i}⊗N_{k}\right)U_{i,k}\rho_{ik}U_{i,k}^{†}\left|\right|\left(I_{i}⊗N_{k}\right)U_{i,k}\left(\rho_{i}⊗\rho_{k}\right)U_{i,k}^{†}))\leq D(U_{i,k}\rho_{ik}U_{i,k}^{†}\left|\right|U_{i,k}\left(\rho_{i}⊗\rho_{k}\right)U_{i,k}^{†}) \end{array}$$

which means that both the Mutual Information and the Holevo Information for identical, separate interactions will be upper bound by the same interaction without any further reduced dynamics on the fragment. From this analysis we gather that, given such local, commuting operations between both subsystems and the same fragments, there is a distinct upper bound to the information that either can share with the environmental fragments. Both of them will be bounded from above by the shared information achieved if the second commuting operation had not occurred, i.e., by the measures from Section 4.1 and Section 4.2.

**Figure 10:**
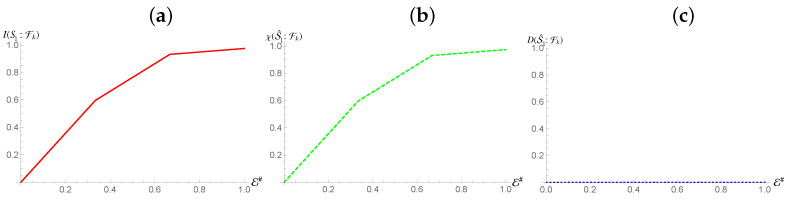
Panel (**a**,**b**): Mutual information (Holevo information) between the whole system and environmental fraction $F_{k}$ for the Hamiltonian model in Equation (14). Panel (**c**): Quantum Discord between the environmental fragments $F_{k}$ and the whole system for the same Hamiltonian model as in panels (**a**,**b**). In all panels, the horizontal axis shows the size of the environment $E^{\#}$, while for all the simulations considered we have taken Jt=10 with *t* the evolution time. The quantities on the vertical axis on all graphs reported int he manuscript are in units of the entropy of the systems of interests.

**Figure 11:**
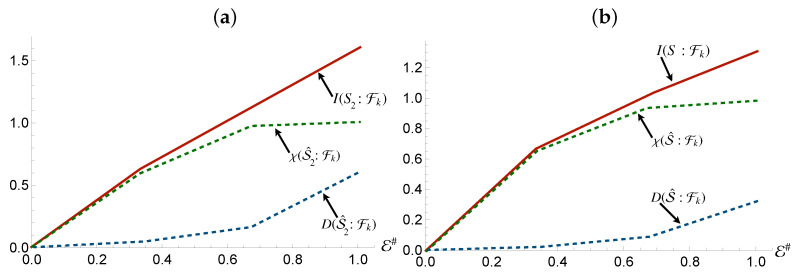
Panel (**a**): Mutual information, Holevo information and Quantum Discord between $S_{2}$ and and environmental fraction $F_{k}$ for the Hamiltonian model in Equation (13). Panel (**b**): Mutual information, Holevo information and Quantum Discord between the environmental fragments $F_{k}$ and the whole system for the same Hamiltonian model as in panels (**a**). In all panels, the horizontal axis shows the size of the environment $E^{\#}$, while for all the simulations considered we have taken Jt=10 with *t* the evolution time.

We observe a staggered, or offset redundancy, of both the Mutual Information, Holevo Information, and Quantum Discord. Figure 10 and Figure 11 show this for the first, second and whole system: in the case of the Holevo Information, the redundancy plateau does emerge, but only for larger fragments (in this case involving two elements or more), which matches up with the size of the system. This is due to the inability of the fragments to increase their entropy enough, or store enough information, to contain that of the state of both $S_{1}$ and $S_{2}$, given both are interacting at the same time, but separately [44].

Mutual Information however, only becomes staggered redundant in the case of the first system. $S_{2}$ and the compound system see an almost linear rise in the Mutual Information as the number of fragments increase. This result is reminiscent of what is found by considering a randomly picked state of a system and its environment, a case addressed explicitly in Ref. [2], where it was shown that such states typically do not display redundant encoding of information on the system. As a result, the addition of more fragments gives a linear rise to the Mutual Information available to the observer. However, the degree of redundancy of the Holevo Information that $S_{2}$ and the whole system share with the fragments is identical to that of the state involving $S_{1}$. This would suggest that, as in the case for $S_{1}$, a larger environment fragments would be more suitable to *accommodate* the information of the subsystems. This leads us to an interesting observation: despite the information not being redundantly encoded, at least in the case of $S_{1}$, it possesses only classical correlations, whilst still remaining non-objective.

The Quantum Discord is, as expected, completely absent from the joint state of the fragments and $S_{1}$, due to the orthogonal nature of the supports, allowing a local projective measurement to reveal all information. Due to this, there should be no information regarding superpositions available at any level in the system as the reduced state at any level will be purely classically correlated. The slight rise for $S_{2}$ and the whole compound reflects the usual behaviour of Quantum Darwinism. When the whole system becomes available, measurements that reveal superpositions of the pointer states become possible, allowing access to quantum information otherwise inaccessible in local fragments.

**Figure 12:**
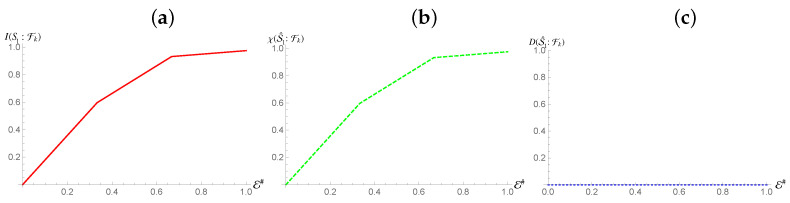
Panel (**a**,**b**): Mutual information (Holevo information) between $S_{1}$ and environmental fraction $F_{k}$ for the Hamiltonian model in Equation (14) given measurements on the fragments. Panel (**c**): Quantum Discord between the environmental fragments $F_{k}$ and $S_{1}$ for the same Hamiltonian model as in panels (**a**,**b**) given measurements on the fragments. In all panels, the horizontal axis shows the size of the environment $E^{\#}$, while for all the simulations considered we have taken Jt=10 with *t* the evolution time. The quantities on the vertical axis on all graphs are in units of the entropy of the systems of interests.

**Figure 13:**
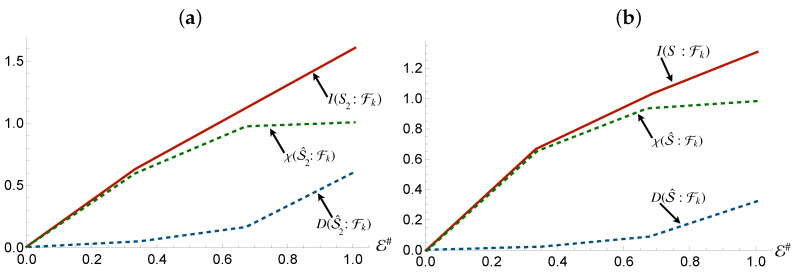
Panel (**a**): Mutual information, Holevo information and Quantum Discord between $S_{2}$ and and environmental fraction $F_{k}$ for the Hamiltonian model in Equation (13) given measurements on the fragments. Panel (**b**): Mutual information, Holevo information and Quantum Discord between the environmental fragments $F_{k}$ and the whole system for the same Hamiltonian model as in panel (**a**), given measurements on the fragments. In all panels, the horizontal axis shows the size of the environment $E^{\#}$, while for all the simulations considered we have taken Jt=10 with *t* the evolution time.

As shown in Figure 12 and Figure 13 the environmental fragments share the exact same ratio of quantum and classical information as the system does. This means that the channel capacity of the system is identical to that of the fragments.

Finally, we wish to see if these interactions have affected the Quantum Discord present between the two subsystems, which was completely absent in the case of measurements on $S_{1}$, and consisted of a fraction of the Mutual Information in the case of measurements on $S_{2}$. After the interactions, we see the Mutual Information for the state of the two subsystems is basically halved. However, in the case of measurements on $S_{2}$, the Quantum Discord becomes only a very small fraction of the total Mutual Information, indicating that after the interaction, almost all the information shared by the two subsystems is classical in nature. The Holevo Information for $\rho_{S_{1}}$ remains unchanged, thus showing that the map between the two is of the discord-breaking form.

### 4.4. Simultaneous Three-Body Interaction

**Figure 14:**
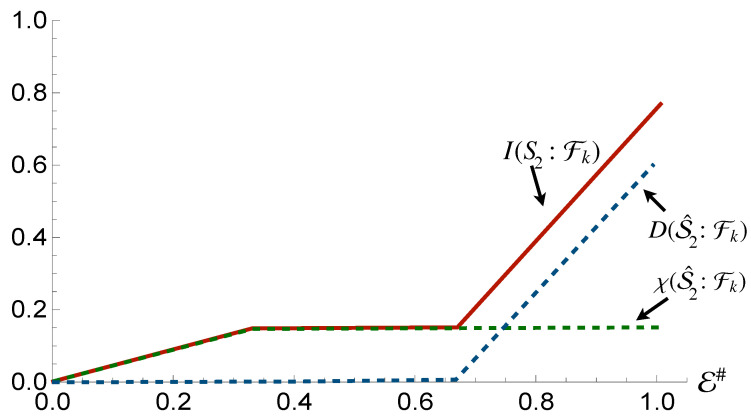
Mutual information, Holevo information and Quantum Discord between $S_{2}$ and environmental fraction $F_{k}$ for the Hamiltonian model in Equation (15). The horizontal axis shows the size of the environment $E^{\#}$, while for all the simulations considered we have taken Jt=10 with *t* the evolution time.

We conclude out analysis by addressing the case of three-body interactions involving the compound system and individual elements of the environment, as illustrated in Equation (15). The Mutual Information and Holevo Information for the state of the $\rho_{S_{1}}$ and environmental fraction take the same values as those achieved when $S_{2}$ mediated the interactions between $S_{1}$ and the environment, as shown in Figure 7. This indicates that an interaction encompassing the whole system is not capable of redundantly encoding any more information regarding $S_{1}$ than a mediated one. No Quantum Discord is present between $S_{1}$ and the environment, confirming that the map is not of the discord creating form. Comparing the results in Figure 14 for $S_{2}$ to those in Figure 8, obtained as a result of Equation (13), we see that there is now also a large deficit to the Mutual Information shared with the fragments, just like in the case of $S_{1}$ for Equation (13). This time, however, the deficit with respect to $S_{2}$ is even larger than that of $S_{1}$. However, the information is completely classical, satisfying Strong Quantum Darwinism once again. There is, in this case, the largest increase of Quantum Discord when the whole environment becomes available, overtaking the Holevo Information for the first time thus far. This suggests that the overall state of the fragments shares almost entirely quantum information with $S_{2}$, although not redundantly.

**Figure 15:**
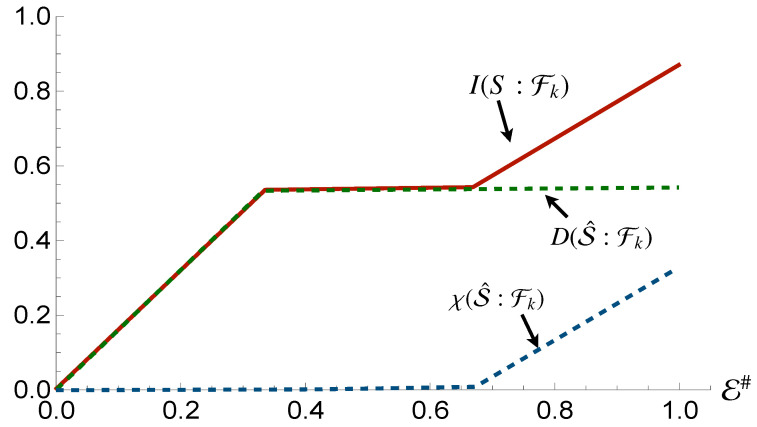
Mutual information, Holevo information and Quantum Discord between the whole system and environmental fraction $F_{k}$ for the Hamiltonian model in Equation (15). The horizontal axis shows the size of the environment $E^{\#}$, while for all the simulations considered we have taken Jt=10 with *t* the evolution time.

Finally, when moving to global measurements of the system, we again see from Figure 15 a behavior in line with the one seen in Figure 9: the encoding of information in this case is redundant, and the nature of Mutual Information completely classical. Quantum information is shared only once we observe the whole environment.

The measurements on the environment yielded no significant change in the amount of Holevo Information. From these results, one would deduce the existence of a distinct upper bound to the degree of redundancy of Mutual Information, Holevo Information, and Quantum Discord between either all the compound system and the fragments, or a subset of the former, when an interaction involves said system. Such an upper bound appears to stem from the lowest value available for the information measures between one of the subsystems comprised in the compound and the fragments, when the interaction has taken place only between that subsystem and the fragments.

## 5. Conclusions

We have considered several models for the interaction between a multipartite environment and a compound system whose elements can share both classical and quantum correlations, aiming at the characterization of the process of information encoding on the system into the environment. We have investigated how different Hamiltonian models affect the phenomenology of information encoding with a specific focus on the possible emergence of redundancy typical of Darwinistic behaviors.

For interactions of the type shown in Equation (13), we have shown that the measures of Mutual Information and Holevo Information between the uncoupled subsystem and the environmental fragments are upper bounded by the values of these quantities calculated over the initial state of the system comprising $S_{1}$ and $S_{2}$. This indicates that initial correlations between subsystems plays a crucial role in the emergence of redundant encoding of information. Due to Equation (A6), this means that the initial values of Holevo Information for the states $\rho_{S_{2}}$ and $\rho_{S_{1}}$ will limit the amount of quantum information that can be found in the state of the environmental fragments. In other words, the higher the initial Quantum Discord, the higher the Quantum Discord that can be shared by the elements of such fragments.

As a consequence, too small initial values of Mutual Information, Holevo Information, and Quantum Discord would result in the inability for mediated redundancy to occur. Similar considerations hold for the case where both subsystems interact with the environment separately, requiring a larger environment to encode the information that would have been more readily encoded via simple local interactions with the subsystems, given an adequate degree of initial correlations. Lastly, we showed that a global interaction between the whole system and the environment can only encode information at best as well as the local interactions do.

Our study pinpoints the intricacies of the interplay between (quantum) correlations, mediated interactions and redundancy of information encoding, highlighting the need for a deeper theoretical framework tacking the transition to classicality and accounting for the features at the core of the study reported here in a structural manner.

## Data Availability

The data presented in this study are available from the authors upon reasonable request.

## Appendix A

We aim at proving the following statement, which is valid within the context of our investigation

(A1) $$I\left(S_{2}:S_{1}\right)\geq I\left(S_{2}:F_{k}\right)$$

**Proof.** In light of invariance of the mutual information under local unitary transformations, we have

(A2) $$I\left(S_{2}:S_{1}\right)=I\left(S_{2}:S_{1},F_{k}\right)=I\left(S_{2}:U\left(S_{1},F_{k}\right)U^{†}\right)$$

with *U* a unitary transformation. By definition, we have

(A3) $$I\left(S_{2}:US_{1},F_{k}U^{†}\right)=R(\left(S_{2},U\left(S_{1},F_{k}\right)U^{†}\right)\left|\right|S_{2}⊗U\left(S_{1},F_{k}\right)U^{†})$$

with $R\left(x||y\right)=\mathrm{tr}[\rho_{x}\left(\log\rho_{x}-\log\rho_{y}\right)]$ the relative entropy between states $\rho_{x}$ and $\rho_{y}$ [36], and where we have emphasized the tensor product nature of states, when due. Due to the monotonicity of relative entropy, we have

(A4) $$\begin{array}{cc} R(S_{2},U\left(S_{1},F_{k}\right)U^{†}||S_{2}⊗U\left(S_{1},F_{k}\right)U^{†}) & \geq R({\mathrm{tr}}_{S_{1}}\left(S_{2},U\left(S_{1},F_{k}\right)U^{†}\right)||{\mathrm{tr}}_{S_{1}}\left(S_{2}⊗U\left(S_{1},F_{k}\right)U^{†}\right))\\ \; & =I(S_{2}:F_{k}), \end{array}$$

thus completing the proof. □

The above steps can also be used to show that

(A5) $$\chi \left({\hat{S}}_{2}:S_{1}\right)\geq \chi \left({\hat{S}}_{2}:F_{k}\right)$$

due to the Holevo Information being just the Mutual Information of the resultant classical-quantum state obtained after the measurement map is obtained, and as such is equivalent to the relative entropy function, and so inherits its properties.

Finally, we aim at proving that

(A6) $$D\left({\hat{S}}_{1/2}:E/F_{k}\right)\leq S\left(\rho_{S_{1/2}}\right)-\chi \left({\hat{S}}_{1/2}:S_{2/1}\right)$$

**Proof.** We use the result from Ref. [26] and a pure state such as $|S_{1},S_{2}E,R\rangle$ to obtain

(A7) $$D\left({\hat{S}}_{1/2}:E/F_{k}\right)=S\left(\rho_{S_{1/2}}\right)-\chi \left({\hat{S}}_{1/2}:S_{2/1},F_{k}R\right)$$

$\chi \left({\hat{S}}_{1/2}:S_{2/1},F_{k}R\right)\geq \chi \left({\hat{S}}_{1/2}:S_{2/1},F_{k}\right)$ due to the monotonicity of relative entropy, giving

(A8) $$D\left({\hat{S}}_{1/2}:E/F_{k}\right)\leq S\left(\rho_{S_{1/2}}\right)-\chi \left({\hat{S}}_{1/2}:S_{2/1},F_{k}\right)$$

We have already shown that $\chi \left({\hat{S}}_{1/2}:S_{2/1}\right)\geq \chi \left({\hat{S}}_{1/2}:F_{k}\right)$ for measurements on the subsystem non involved with the interaction, therefore

(A9) $$D\left({\hat{S}}_{1/2}:E/F_{k}\right)\leq S\left(\rho_{S_{1/2}}\right)-\chi \left({\hat{S}}_{1/2}:S_{2/1}\right)\leq S\left(\rho_{S_{1/2}}\right)-\chi \left({\hat{S}}_{1/2}:F_{k}\right).$$

□
